# Maternal Serum and Placental Tissue Expression of PLGF and sFlt-1 in Placenta Accreta Spectrum: Correlation With International Federation of Gynecology and Obstetrics (FIGO) Grading of Disease Severity

**DOI:** 10.7759/cureus.112581

**Published:** 2026-07-13

**Authors:** Sheetal Rani, Deepthi Nayak, Mamta Singh, Amrita Ghosh Kar, Usha Rani Behera

**Affiliations:** 1 Obstetrics and Gynaecology, Institute of Medical Sciences, Banaras Hindu University, Varanasi, IND; 2 Pathology, Institute of Medical Sciences, Banaras Hindu University, Varanasi, IND

**Keywords:** angiogenesis, hemorrhage, placenta accreta spectrum, plgf, sflt-1

## Abstract

Introduction: Angiogenesis is a central process in the development of the placenta. This balance between pro-angiogenic and anti-angiogenic factors in the decidua can impact the depth of invasion of the placental villi. This study aimed to understand the relationship between serum PLGF (placental growth factor) and sFlt-1 (soluble fms-like tyrosine kinase-1) levels and placental tissue expression of PLGF and sFlt-1 in placenta previa and placenta accreta spectrum (PAS) and to better understand the local and/or systemic role of PLGF and sFlt-1 in the different FIGO PAS grades.

Methodology: A case-control study was conducted in the Department of Obstetrics and Gynecology, Institute of Medical Sciences, Banaras Hindu University, a tertiary care hospital in Northern India, between September 2024 and February 2026. Women with normal placental implantation diagnosed on third trimester ultrasound (USG) were controls, and women with USG showing evidence of placenta previa or PAS were cases. The serum levels of sFlt-1 and PLGF and placenta bed immunohistochemical (IHC) of sFlt-1 and PLGF were analysed between the two groups and between different FIGO grades of PAS. Analysis was done with IBM SPSS Statistics.

Results: Fifty women with normal placental implantation (control) and 50 women with placenta previa without invasion or PAS (cases) (placenta previa without invasion -17, FIGO grade 1 PAS-5, grade 2 PAS-12, grade 3 PAS-16) were included in the study. The median age (cases median age = 30, controls = 24, p < 0.001), history of prior caesarean delivery (42 (84%) vs. 6 (12%); p < 0.001) and history of placenta previa in prior pregnancies (21 (42%) vs. 0; p < 0.001) of the cases was significantly higher among the cases as compared to the controls. Serum sFlt-1 (median 5,350 vs. 7,550; p < 0.001) and serum PLGF values (2,550 vs. 5,150, p < 0.001) were lower among cases as compared to controls; there was no difference in the placenta IHC score for sFlt-1 (5 vs. 5; p = 0.770) and PLGF (2 vs. 2; p = 0.106) between groups. In the subgroup analysis of the cases, there was no significant difference between placenta previa without invasion and various grades of PAS with regard to sflt-1, PLGF, sFlt-/PLGF ratio, and placental IHC score for sFlt-1 and PLGF.

Conclusion: Angiogenic markers, such as PLGF and sFlt-1, are useful adjunctive biomarkers for identifying abnormal placentation. However, their role is limited to detection rather than grading of PAS.

## Introduction

Placenta accreta spectrum (PAS) disorders represent a group of severe placental implantation abnormalities characterized by defective decidualization and abnormal trophoblastic invasion into the uterine wall. In PAS, the villi can be directly attached to the myometrium and invade further into the uterus, reaching the serosal surface and the surrounding organs, most commonly the bladder [1]. This aberrant placentation disrupts the normal placental-uterine interface and is associated with catastrophic obstetric haemorrhage and maternal morbidity, such as disseminated intravascular coagulation, injury to adjacent organs, and need for intensive care unit admission [2].

The global incidence of PAS has risen over the past four decades due to the contemporary increase in caesarean delivery rates. The incidence of PAS ranged from 0.01 to 1.1%, according to a recent meta-analysis of population-based research, with a combined prevalence of 0.17% [3]. The incidence of PAS in our institute is 10.72 per thousand deliveries [4]. This uptrend is observed equally across developing and developed countries. Rising institutional delivery rates and increasing cesarean section prevalence, particularly in urban and semi-urban Tier-II centres, have contributed to a growing number of PAS cases presenting to tertiary referral hospitals [5,6]. 

The International Federation of Gynecology and Obstetrics (FIGO) has standardized the classification of PAS disorders into three clinicopathological grades based on the depth of invasion, and this has brought uniformity in the diagnosis, reporting, and management of PAS across clinical settings [1]. Ultrasonography is the primary screening modality. However, diagnostic accuracy varies significantly based on operator expertise and gestational age at evaluation [7]. Magnetic resonance imaging (MRI) is frequently used as an adjunct, particularly in suspected posterior placentation. One of the major limitations in current PAS management is the absence of reliable systemic biomarkers that can predict disease severity or depth of invasion.

Angiogenesis is a central process in placental development, ensuring adequate uteroplacental blood flow to support fetal growth. Placental growth factor (PLGF) is a pro-angiogenic member of the vascular endothelial growth factor (VEGF) family and plays a crucial role in trophoblast proliferation, vascular remodelling, and placental perfusion [8]. Soluble fms-like tyrosine kinase-1 (sFlt-1) is an anti-angiogenic protein that acts as a decoy receptor by binding VEGF and PLGF, thereby reducing their bioavailability [9]. The balance between PLGF and sFlt-1 is essential for maintaining normal placental vascular homeostasis. Placental tissue from PAS cases demonstrates differential expression of VEGF, PLGF, and their receptors, indicating dysregulated angiogenic signalling within invasive trophoblast populations [10]. 

The current literature highlights a significant knowledge gap regarding the relationship between systemic angiogenic factor levels and local placental tissue expression in PAS. This study aimed to understand the relationship between serum PLGF and sFlt-1 levels and placental tissue expression of PLGF and sFlt-1 in placenta previa and PAS and to better understand the local and/or systemic role of PLGF and sFlt-1 in the different FIGO PAS grades.

## Materials and methods

This was an analytical case-control study conducted in the Department of Obstetrics and Gynecology, Institute of Medical Sciences, Banaras Hindu University, a tertiary care hospital in Northern India (eastern Uttar Pradesh), between September 2024 and February 2026. Institutional Ethics Committee approval was obtained before the commencement of the study, and the study was conducted in full compliance with the Declarations of Helsinki (IMS/IEC/2024/7412). All pregnant women with a third-trimester ultrasound diagnosis of placenta previa (leading edge of the placenta within 2 cm from the internal Os or the placenta completely covering the internal os) [11] with or without suspicion of invasion planned for elective caesarean delivery were recruited as cases. Women with suspicion of PAS on ultrasound based on the FIGO criteria [1] were subjected to MRI testing for confirmation. The final confirmation and grading were based on the intraoperative and histopathological findings. Women with normal placental localisation undergoing elective caesarean delivery for various other obstetric indications were recruited as controls. Women with multiple gestations, fetal growth restriction, hypertensive disorders of pregnancy, obesity, and metabolic disorders complicating pregnancies were excluded from the study. Participants were recruited using a non-probability convenience sampling technique, with equal allocation of 50 cases and 50 controls. Cases comprised those with abnormal placentation, such as placenta previa or PAS. Among the cases, women were further divided into those with placenta previa alone without invasion, FIGO grade 1/grade 2/grade 3 as per the FIGO classification for PAS [1]. Management of the cases was done as per the standard protocol, i.e., in women with placenta previa without caesarean, a standard lower segment caesarean section was planned. In women with PAS, classical caesarean section was done, and if the placenta spontaneously delivered with no haemorrhage, hysterotomy was closed; if no separation of the placenta was possible with intrapartum haemorrhage, caesarean hysterectomy was undertaken.

All women were recruited after obtaining written informed consent. The demographic details and clinical details of the patients were obtained by the interview method. The imaging details and labour and postoperative details were retrieved from the medical records of the patients. Then, 5 mL of blood was retrieved by venepuncture from all the women recruited for the study just prior to delivery. After collection, the sample was immediately centrifuged, and the separated serum was stored at -20°C. Serum concentrations of sFlt-1 and PLGF were analysed using the human sVEGFR-1/sFlt-1 ELISA kit, Picokine, human PLGF ELISA kit, and PicoKine™ kit, respectively, by quantitative sandwich enzyme immunoassay technique. These kits use a quantitative sandwich enzyme immunoassay technique. The minimum detectable doses in the assays for sFlt-1and PLGF were <30 and <1 pg/ml, respectively, with the intra-assay precision of the sFlt-1 and PLGF reagent kits equal to 4.1-7.8% and 5.6-7.9%, respectively, while the inter-assay precision was 4.7-9.7% and 5.6-9.5%, respectively. All laboratory procedures were conducted by an independent technician in the Clinical Pathology Laboratory of our hospital to ensure analytical objectivity. In women who underwent caesarean section alone, placental bed tissue along with the placenta was obtained during caesarean section, and immunohistochemical staining was performed for sFlt-1 and PLGF on the tissue. For women who underwent caesarean hysterectomy, the entire specimen of the uterus with placenta was sent for histopathological examination for the confirmation of clinical PAS diagnosis and immunohistochemical staining. Two parameters were evaluated: a) percentage of stained trophoblast cells (score 1: 0-10%, score 2: 10-50%, score 3: ≥50%) and b) staining Intensity (score 1: weak, score 2: moderate, score 3: strong). The final expression of the score is calculated by

\[ FS = PS \times IS \], where FS: final score, PS: percentage score, and IS: intensity score (range: 1-9).

This semi-quantitative scoring method provided objective grading of tissue expression. The grading was done by one independent pathology consultant with more than 10 years of experience in this field.

The data collected were entered into IBM SPSS Statistics for Windows, version 30.0 (released 2024, IBM Corp., Armonk, NY) for statistical analysis. Normality of the data was assessed by the Shapiro-Wilk test, and with a p-value <0.05, the dataset was considered non-normally distributed. Categorical data are presented as percentages and continuous data as mean with standard deviation or median with range. Mann-Whitney U was used to compare continuous variables between cases and controls, whereas the Kruskal-Wallis test was used to compare the continuous data between the subgroups of cases. Spearman’s rank correlation test was used to determine the association between serum sFlt-1 and PLGF with those of placental staining and FIGO grade of PAS. To identify factors independently associated with increasing severity of PAS, an ordinal logistic regression model was constructed using case subgroups (placenta previa, FIGO grade 1, FIGO grade 2, and FIGO grade 3) as the dependent variable. Odds ratio (OR) with 95% confidence interval was reported. A p-value of < 0.05 was considered significant.

## Results

The total study sample included 100 women, comprising 50 cases and 50 controls. Within the case group, the diagnostic categories were placenta previa without invasion (n = 17, 34%), FIGO grade I (n = 5, 10%), FIGO grade II (n = 12, 24%), and FIGO grade III (n = 16, 32%). The basic demographic profile of the cases and controls is depicted in Table 1. The median age of the cases is 30 years and of the controls is 24 years, and the difference could be due to more primiparous women in the control group.

**Table 1:** Demographic characteristics of cases and controls *Results depicted as median and Interquartile range; the rest as frequency (percentage). *Comparison between medians by Mann-Whitney U test; # variables are compared with Fisher's exact test; ^ variables are compared by chi-square test). BMI: body mass index, UTI: urinary tract infection

Variable		Cases (placenta previa/PAS); N = 50	Control (normal placentation); N = 50	P -value
Age (years)*		30 (27-32)	24 (23-26)	<0.001
Age group (years)	<24	3 (6%)	26 (52%)	
	25-35	44 (88%)	24 (48%)	
	>35	3 (6%)	-	
BMI (kg/m^2^)*		21.88 (23.40-24.50)	23.85 (23.42-24.58)	0.917
Socioeconomic status ^#^	Upper middle	6 (12%)	-	0.028
	Lower middle	26 (52%)	25 (50%)	
	Upper lower	18 (36%)	25 (50%)	
Parity^	Primigravida	5 (10%)	16 (32%)	0.011
	Multigravida	45 (90%)	34 (68%)	
Previous caesarean section^		42 (84%)	6 (12%)	<0.001
Prior uterine surgeries/procedures	Dilatation and curettage	16 (32%)	2 (10%)	
	myomectomy	5 (4%)	-	
h/o placenta previa in previous pregnancy ^#^		21 (42%)	-	<0.001
h/o infection during pregnancy (UTI or vaginitis) ^		16 (32%)	9 (18%)	0.165
Gestational age at delivery (weeks) ^#^	28-33+6	15 (30%)	-	<0.001
	34-36+6	25 (50%)	(8%)	
	37-40	10 (30%)	46 (92%)	

Among the cases, 29 (58%) underwent caesarean hysterectomy, 21 (42%) underwent caesarean section. Further subgroup analysis showed, One patient with FIGO grade 1 PAS (1, 20%), 12 women with FIGO grade 2 (12, 100%) and 16 women with FIGO grade 3 PAS (16, 100%) underwent caesarean hysterectomy. All the controls were delivered by caesarean section and none underwent hysterectomy. Two of the women with FIGO grade 3 PAS (2, 12.5% of grade 3 PAS and 4% of women with abnormal placentation) succumbed to hypovolemic shock with disseminated intravascular coagulation

Comparison of sFlt-1, PLGF and sFlt-1/PLGF ratio between cases and controls by Mann Whitney U test showed that pregnancies with abnormal placentation demonstrated significantly lower PLGF concentration and lower sFlt-1 levels as compared to women with normal placentation. The ratio of sFlt-1/PLGF was significantly higher among cases. All the differences were associated with large effect size (0.767-0.996). In contrast, immunohistochemical (IHC) assessment of trophoblast showed no significant difference between the groups with regards to the staining proportion, intensity and the final score. (Table 2)

**Table 2:** Comparison of serum markers and placenta markers on IHC between groups Mann Whitney U test. All values are measured as median (minimum-maximum) value. PLGF- placental growth factor, sFLT-1- soluble fms like tyrosine kinase-1, IHC- immunohistochemistry

Variable	Cases (median) N=50	Control (median) n=50	P value	Effect size
Age	30 (27-32)	24 (23-26)	<0.001	-0.76680
Serum PLGF (pg/ml)	2550 (800-4200)	5150 (4000-6000)	<0.001	0.99560
Serum sFlt-1 (pg/ml)	5350 (2600-7200)	7550 (6200-9000)	<0.001	0.95280
Serum sFlt-1/PlGF ratio	2.4 (1.2-3.8)	1.5 (1.2-2)	<0.001	-0.82160
Percentage of sFlt-1 stained trophoblast	30 (20-70)	30 (15-75)	0.597	-0.06120
sFlT-1 staining intensity	2 (1-3)	2 (1-3)	0.429	0.08640
sFlt-1 final IHC score	5 (3-9)	5 (2-9)	0.770	-0.03360
Percentage of PLGF stained trophoblast	37.5 (20-80)	35 (20-75)	0.992	0.00160
PLGF staining intensity	2 (1-3)	2 (1-3)	0.119	0.16960
PLGF final IHC score	5 (2-9)	5 (3-9)	0.106	0.18440

The values of serum markers between the various subcategories among the cases are depicted in Table 3. Kruskal-Wallis test showed no significant difference in serum sFlt-1, PLGF, sflt-1/PLGF ratio, or placental IHC for the above factors between the subgroups of cases. Placental PLGF demonstrated a trend toward significance corresponding to a moderate size effect. Spearman rank correlation analysis demonstrated no significant association between the PLGF IHC score and FIGO grade of PAS (ρ = -0.156, p = 0.278). 

**Table 3:** Comparison of serum markers and placenta IHC markers between different FIGO grades of PAS Kruskal-Wallis test. All values are measured as median (minimum-maximum) values. PLGF: placental growth factor, sFLT-1: soluble fms-like tyrosine kinase-1, IHC: immunohistochemistry, FIGO: International Federation of Gynecology and Obstetrics, PAS: placenta accreta spectrum

Variable	Placenta previa without invasion (n = 17)	FIGO grade 1 PAS (n = 5)	FIGO grade 2 PAS (n = 12)	FIGO grade 3 PAS (n = 16)	H (df = 3)	P-value	ε^2^
Serum PlGF (pg/ml)	2500 (800-4200)	2200 (900-3800)	2400 (1000-3700)	2650 (850-4100	0.187	0.980	0.004
Serum sFlt-1 (pg/ml)	5500 (3000-7000)	4800 (2900-6500)	5100 (2600-7200)	5950 2800-7100)	0.206	0.977	0.004
Serum sFlt-1/PlGF ratio	2.4 (1.2-3.8)	2.1 (1.6-3.2)	2.4 (1.7-3.0)	2.5 (1.5-3.3)	0.229	0.973	0.005
sFlt-1 final IHC score	5 (3-9	5 (3-6)	5 (3-9)	5 (3-9)	1.571	0.666	0.032
PLGF final IHC score	5 (2-9)	8 (5-9)	3.5 (2-9)	5 (2-9)	7.370	0.061	0.150

Multivariate ordinal logistic regression was performed with maternal age, serum PLGF, placental sFlt-1 IHC score, and placental PLGF IHC score across the subgroups of cases, which showed that history of previous caesarean section (aOR = 30.09, 95% CI = 26.52-34.15, p value <0.001) and maternal age (aOR = 1.19, 95% CI = 1.05-1.34, p = 0.004) showed positive correlation with higher FIGO grade. Maternal serum PLGF (aOR = 1, 95% CI = 0.999-1.001, p = 0.474), placental sFlt-1 IHC score (aOR = 0.80, 95% CI = 0.61-1.06, p = 0.123) and history of placenta previa in prior pregnancies (aOR = 2.34, 95% CI = 0.76-7.23, p = 0.138), gestational age at delivery (sampling) (aOR 0.58, 95% CI = 0.25-1.32, p = 0.191) were not significant predictors of PAS severity. Placental PLGF IHC score demonstrated an independent inverse association between the PAS grade (aOR = 0.69, 95% CI = 0.51-0.93, p = 0.016), indicating lower PLGF expression with increasing severity of invasion. The model demonstrated moderate explanatory ability (McFadden’s pseudo-R2 = 0.194).

## Discussion

There were 50 women with normal placental implantation (control) and 50 women with placenta previa without invasion or PAS (cases) included in the study. The median age, history of prior caesarean delivery, and history of placenta previa in prior pregnancies of the cases were significantly higher among the cases as compared to the controls. All women with FIGO grade 2/3 PAS underwent caesarean hysterectomy. Serum sFlt-1 and serum PLGF values were lower among cases as compared to controls; however, there was no difference in the placenta IHC score for sFlt-1 and PLGF between groups. In the subgroup analysis of the cases, there was no significant difference between placenta previa without invasion and various grades of PAS with regard to sflt-1, PLGF, sFlt-/PLGF ratio, and placental IHC score for sFlt-1 and PLGF. Multivariate ordinal regression analysis showed advanced maternal age and history of prior caesarean delivery having a positive correlation with higher grade of FIGO, and low placental PLGF IHC score showed a negative correlation with higher grade of PAS.

PAS is a consequence of abnormal penetration of the trophoblastic tissue beyond the decidua basalis into the surrounding pelvic organs. This can lead to catastrophic obstetric haemorrhage, need for caesarean hysterectomy, ureteric/bladder injuries, fetal prematurity, maternal mortality, and morbidity. The exact pathophysiology is unclear; however, various risk factors are identified for the development of this condition, such as advanced maternal age, prior caesarean delivery, prior placenta previa, previous uterine surgeries like myomectomy or curettage, and pregnancy with assisted reproductive techniques [12]. The same was observed in the present study. Previous caesarean delivery was found to be associated with a risk of higher grades of PAS in the present study (aOR = 30.09, 95% CI = 26.52-34.15, p-value <0.001); however, the higher aOR could be due to the smaller sample size and almost all of the higher FIGO grade PAS women having a history of previous caesarean delivery. Damage to the decidua during caesarean section and the hypoxic environment created by the scar tissue could be the trigger for the invasive trophoblastic behaviour [13].

Numerous regulatory molecules are identified at the maternal-placental interface that influence the depth of invasion of the placental trophoblast, which finally depends on the balance between the pro-angiogenic (such as vascular endothelial growth factor (VEGF) and PLGF) and anti-angiogenic molecules (such as sFlt1 and soluble endoglin) [14]. Disturbance in this balance creates a proangiogenic environment. Lower serum levels of sFlt-1 are reported in women with PAS across various studies. Lower levels of sFlt-1 will enable VEGF to act on its receptor, increasing the neovascularisation, compounded by lower levels of natural killer cells, allowing for the deeper invasion of the trophoblastic tissue [15]. PLGF is critical for placental angiogenesis and normal vascularisation. Alteration in the levels of PLGF can lead to altered angiogenesis and abnormal trophoblastic invasion.

We have observed lower levels of sFlt-1 and PLGF in the serum among women with PAS as compared to those of the controls, consistent with studies by Alessandrini L et al. and Yusrawati et al. [16,17]. A meta-analysis on serum biomarkers among women with PAS showed that serum sFlt was consistently low in all PAS cases across studies; however, the levels of PLGF were inconsistent, with some showing higher levels, some showing the opposite, and some finding no significant difference among women with PAS. This could possibly be due to the difference in the degree of invasion of the placenta, gestational age at sampling, and biomarker assay used [14].

Although the serum levels of sFlt-1 and PLGF were lower among cases as compared to controls, there was no significant difference in the placental expression of sFlt-1 and PLGF in the present study. In the subgroup analysis, the placental PLGF IHC score was higher in FIGO grade I, which could have contributed to the negative relationship between the placental PLGF and severity of PAS grade in logistic regression analysis. This could be due to the extremely small sample size in this group and needs to be further evaluated with a bigger sample size to establish significance. A study by Alessandrini L et al. demonstrated a non-significant difference in placental sFlt and PLGF staining between placenta previa and various FIGO grades of PAS. On evaluating patients after 34 weeks, they observed that placental expression of PLGF increased and placental sFlt-1 decreased with increasing FIGO grade of PAS, which was also reported by Arakaza et al. [16,18].

This study has numerous limitations. First, the sample size within some FIGO subgroups, particularly FIGO I, was small. This reduces statistical power and increases the possibility that true differences across grades may have been missed. Second, placental tissue assessment was based on semiquantitative IHC scoring, which is inherently subject to sampling variability and observer-dependent interpretation. Because PAS lesions are often focal and irregular, a limited tissue section may not represent the full biological behaviour of the placental bed. Third, gestational age differed substantially between cases and controls, with cases being delivered earlier overall. Since serum biomarker levels vary across gestation and sampling for serum biomarkers was done just before delivery, this may have influenced the actual value and group comparisons. This study is a novel study assessing both systemic and placental biomarkers in PAS, and the entire disease spectrum is included.

## Conclusions

Serum levels of sFLT-1 and PLGF are significantly lower among women with PAS; however, placental levels of the same molecules remain unchanged between cases and controls. Maternal age and history of previous caesarean section are independent risk factors for a higher degree of PAS. The serum and placental biomarkers did not vary significantly between various grades of PA. Findings of this study indicate that PLGF and sFlt-1 are useful adjunctive biomarkers for identifying abnormal placentation but not the grade of PAS; however, due to the small sample size in the present study, research with a larger sample size will be needed to further confirm these findings.
